# Feasibility and Effectiveness of a New Short-Term Psychotherapy Concept for Adolescents With Emotional Dysregulation

**DOI:** 10.3389/fpsyt.2020.585250

**Published:** 2021-01-21

**Authors:** Andrea Dixius, Eva Möhler

**Affiliations:** ^1^SHG Klinik für Kinder- und Jugendpsychiatrie, Psychotherapie, Psychosomatik, Saarbruecken, Germany; ^2^Saarland University Hospital, Homburg, Germany

**Keywords:** emotion regulation, stress, trauma, resilience, psychotherapy

## Abstract

**Background:** The ‘Stress-Trauma-Symptoms-Regulation-Treatment’ (START) is an innovative manualized short-term treatment program for stabilization and stress resilience in emotionally dysregulated adolescents, based on an approach of stress and management and emotional regulation. The current pilot trial aims to assess the feasibility and effectiveness of the START intervention program for improvement of emotion regulation.

**Methods:** Sixty-six adolescents between the age of 13–18 years admitted to a psychiatric unit for acute emotional or behavioral dysregulation took part in the START program for 5 weeks in an open group setting with two sessions per week (70 min/session). Before treatment, we assessed a history of adverse experience with the Child and Adolescent Trauma Screen (CATS) and the Child Posttraumatic Cognitions Inventory (CPTCI). Before and after treatment, the participants completed the FEEL-KJ, a self-report screening instrument of emotional regulation and coping strategies.

**Results:** A large proportion of this sample had experienced traumatic events based on the CATS (75%) and the CPTCI (82%). The mean FEEL-KJ score significantly decreased after the intervention (*d* = −0.248, *p* = 0.037), while no difference was observed with regard to mean level of adaptive emotion regulation strategies (*d* = 0.202, *p* = 0.207). A positive effect of the intervention was observed on three components of the adaptive FEEL-KJ scale: accepting *(d* = 0.289, *p* = 0.08), forgetting (*d* = 0.271, *p* = 0.049) and dealing with anger (*d* = 0.309, *p* = 0.034).

**Conclusion:** START demonstrates preliminary evidence for improvement in emotional dysregulation after a 5-weeks course of treatment. Therefore, this short-term intervention can possibly be regarded as a tool to improve emotional stability in children with a high load of trauma-related psychopathology. The results are promising and warrant future studies, specifically randomized controlled trials on the effectiveness of START for strengthening resilience at-risk-populations.

## Introduction

The ability to regulate emotional responses to events, situations, and experiences is central to mental health (1). Mood Regulation successfully contributes to higher self-effectiveness, a central element for identity development in adolescents (2). Emotional regulation (ER) strategies as well as a sense of self-worth are fundamental elements of resilience (3–6). Dysfunctional strategies for ER such as self-harming behavior, impulsive behavior, or substance abuse are often used for managing internal tension and negative emotions (7–12).

Incidence of mental disorders, specifically emotional disorders (13) in children is continuously increasing (14). Emotional dysregulation (ED) is associated with diverse forms of childhood psychiatric disorders and symptoms like post-traumatic stress disorder (PTSD), oppositional defiant- and conduct disorders (ODD and CD), borderline personality disorders (BPD), self-injurious behavior and suicidality and attention deficit hyperactivity disorder (ADHD).

ED represents a major health risk reported in about 5% of children and adolescents (15, 16). In clinical settings, dysregulation problems are especially prominent (17), occurring in 26–31% of children admitted to child and adolescent psychiatric clinics or mental health facilities. The occurrence of typical phenomena associated with ED has estimates of about 45 % in child psychiatric patients between 6 and 18 years (12).

Some data indicate a potential influence of early life stress on ED (18, 19). Adolescents without support, such as unaccompanied refugee minors without the protection of their parents or important caregivers are particularly affected and exposed to a greater risk of developing psychiatric disorders related to ED (20–23). A significant proportion of dysregulated adolescents are reported to have been exposed to successive periods of severe stress or trauma (24). Sequential traumatization in this regard is as a succession of traumatizing events maintaining severity of the psychological and physical consequences (25–27). Typical symptoms after experiencing maltreatment include hypervigilance and hyperarousal potentially accompanied by impulsive behavioral problems, and a reduced ability to verbally express emotional experience (28–30). Also it is known that children and adolescents suffering from persistent stress have a high risk of developing mental health problems (18, 24, 31–38).

Emotional stress has been shown do put the body under “permanent alarm” (32, 39, 40). Children and adolescents are at high risk of developing acute stress disorders, adjustment disorders, and also PTSD, grief, and mood disorders (34, 41–45).

Felitti et al. (24) described the relationship of health risk in adulthood of exposure to childhood emotional, physical, or sexual abuse and household dysfunction in a large sample during childhood.

Many afflicted adolescents have been reported to avoid problematic and trauma-associated emotions by use of escape strategies such as dysfunctional behaviors (7, 39, 46–48). Maladaptive ED strategies are thought to be risk factors and connected with the development of mental disorders including self-injurious behavior, substance use, pre-suicidal acts, and impulsive acts (32, 48–52).

Summing up, ED and the experience of traumatic events have repeatedly been described to play a central role in the development of mental disorders and in the development and maintenance of trauma-specific symptoms (49, 53–56). Twenty to 50% of people who have had traumatic experiences show PTSD, but also other child psychiatric conditions can be observed (57). Interventions strengthening stress resilience and emotion regulation seem to be of crucial importance in adolescents at risk.

Stress management has proven to be of importance for young children helping them cope with stressful situations (31, 58, 59). Specifically, low threshold interventions can be used effectively by children and adolescents to reduce stress and regulate emotions also by promoting self-efficacy (60, 61).

Furthermore, early interventions have been described to prevent the increase of trauma symptoms and psychopathology (4, 62, 63). For adaptive emotional regulation, skills such as those used in dialectic behavioral therapy (8, 64, 65, 73) are recommended.

However, the intellectual and motivational requirements of dialectic behavioral therapy exclude a patient population with shorter attention span and/or more unstable therapy commitment, as frequently presenting in acute child psychiatric clinics (66). Therefore, a low threshold program was designed to meet the acute treatment needs of emotionally highly unstable adolescents.

START (Stress-Traumasymptoms-Arousal-Regulation Treatment) is a manualized short-term (5 weeks) treatment concept of stabilization and emotional regulation for extremely stressed adolescents and also for minor refugees. The intervention was constructed to offer adolescents, who are unmotivated or too unstable for Dialectical Behavior Therapy (DBT), reprocessing treatment, or long-term psychotherapy conditions, a first aid for crisis management and emotional stabilization (67). The program can be used for adolescents with diverse cultural backgrounds and repeatedly severe emotional distress, as a low-threshold training program to manage emotional dysregulation including skills to reduce self-harming and impulsive aggressive behavior. The shortness of the treatment program was deliberately planned, as the motivation to seek treatment, adherence, and compliance is often fragile in adolescents (68). Especially adolescents with traumatic experiences as a very vulnerable group are presumed mistrustful and not particularly eager to be kept in therapy for longer periods of time. Therefore, a program not requiring a commitment for longer than 5 weeks seemed to be necessary for acute clinical purposes.

The intervention designed and evaluated in this study places special emphasis on the use of adaptive regulation strategies for acute crisis management and ED (69). In the first step, the short therapeutic program START was developed by Dixius and Möhler (70, 71) and originally conceived for the work with refugee minors only. Minor refugees appear to be a particularly vulnerable group for the development of mental disorders with a range from 20 to 82% (23, 72). In the second step, the intervention was clinically applied in the general population of an acute child and adolescent ward. The contents of the short-term therapy concept integrate and adapt elements and skills for mindfulness, stress regulation, emotion regulation, and self-care from Dialectic Behavioral Therapy (DBT) (11, 64, 65, 73, 74). Easy—to—apply and stimulus rich skills are postulated to improve motivation and emotion regulation capacities. Furthermore, the therapy concept integrates and adapts elements and skills for relaxation and stabilization techniques from psycho-trauma therapy, especially from the Trauma focused-Cognitive Behavioral Therapy (Tf-CBT) (9). As part of mindfulness exercises, the therapy concept includes implicitly bilateral stimulation from Eye Movement Desensitization and Reprocessing (EMDR) (75). Stress disturbs the cooperation of the brain hemispheres—under this aspect these exercises are designed to support the processing of stress (76).

This project examines the hypothesis that the 5-week treatment course of START is able to improve emotional regulation in adolescents referred to acute treatment for suicidal or highly aggressive behavior of mixed cultural backgrounds.

## Methods

A total sample of 66 patients participated in the 5-week START study at an inpatient psychiatric unit.

### Sample

The sample consisted of 17 male and 49 female patients aged between 13 and 18 years (mean value 14,85; *SD* = 1.57) referred to acute inpatient treatment for suicidal or aggressive behavior.

Adolescents with the origin in the following countries were included: Germany (79 %), Afghanistan (9 %), Syria (8%), Poland (2%,) Pakistan (2 %), Gambia (2%).

The study inclusion procedure consists of a consultation with a child and adolescent psychiatrist regarding indication and motivation for treatment and to deliver information about the intervention as well as informed consent. After informed consent, adolescents are assessed for trauma with the Child and Adolescent Trauma Screen (CATS) and the Child Posttraumatic Cognitions Inventory (CPTCI) before treatment. Emotional regulation was measured with the FEEL-KJ scale before and after treatment.

Inclusion criteria were:

- Age 13–18
- Acute referral for suicidal or highly aggressive behavior
- Voluntary participation

Exclusion criteria were:

- Diagnosis of schizophrenic or affective psychosis
- Acute intoxication

The inclusion criteria in this study were not based on diagnoses but on symptoms of ED, such as suicidal behavior or aggressive behavior. Criteria for exclusion were psychoses and acute substance intoxication. Inclusion criteria were based on admission in acute crises for dysregulated emotions, not a diagnosis, nevertheless, the majority of the diagnoses consisted of a complex combination of response to severe stress and PTSD, adjustment disorder, depressive episodes, or borderline disorder. Confirmation of these ICD-10-diagnoses follows a clinical routine work flow with standardized diagnostic procedures specific for the disorder in question, such as e.g., DISYPS III, Conners Scales 3 for ADHD etc.

Drop-out occurred in one case, due to lack of motivation of the participant. The participants with missing information in a questionnaire were excluded from the analysis of this questionnaire.

## Instruments

### CATS: Child and Adolescent Trauma Screening

Sachser et al. (77) assesses the occurrence and impact of traumatic events with a 15-item event scale. In case of the existence of a potentially traumatic event, the traumatic impact is assessed on a 20-item scale. A cut-off of 21 is a presumed indicator of post-traumatic stress. Reliability has been reported to range between 0.88 and 0.94 The convergent-discriminant validity pattern showed medium to strong correlations with measures of depression (*r* = 0.62–82) and anxiety (*r* = 0.40 −0.77) and low to medium correlations with externalizing symptoms (*r* = −0.15–0.43) for participants within informants in all language versions.

### CPTCI-25: Child Post-traumatic Cognitions Inventory – 25

The CPTCI is a self-report questionnaire. This Inventory assesses post-tramatic cognition on a 25-item scale (78).

Clinically relevant trauma is postulated at a score between 46 and 48. Posttraumatic psychopathology of clinical relevance is given at a score above 49. Each item is rated on a four-point Likert scale: “do not agree at all” (1 point), “do not agree a bit” (2 points), “agree a bit” (3 points), and “agree a lot” (4 points). Internal consistency of the scale was Cronbach's Alpha: 0.86–0.93 and Retest-Reliability 0.72–0.78.

### FEEL-KJ: Questionnaire for the Assessment of Emotional Regulation in Children and Adolescents

The questionnaire (79) quantifies 15 strategies for emotional regulation and regulation of the specific emotions (all of which are multi-dimensional and specific to a certain emotion): anxiety, sadness, and anger. In two secondary scales, this instrument identifies seven adaptive and five maladaptive emotional regulation strategies. Adaptive strategies include the following sub-scales: problem-oriented acting, distraction, mood improvement, acceptance, forgetting, cognitive problem solving, dealing with fear, anger, and grief. Maladaptive strategies include the sub-scales: giving up, aggressive behavior, self-devaluation, withdrawal, perseveration, and also dealing with anxiety, anger, and grief. The additional scales are composed of social support, expression, and emotional control.

Internal consistency of the 15 scales ranges between α =0.69 and α = 0.91. Secondary scales show a consistency of α = 0.93 (adaptive strategies) and α = 0.82 (maladaptive strategies). Retest-Reliability (6-week-stability) ranges between *r*_*tt*_ = 0.62 and *r*_*tt*_ = 0.81, and for the secondary scales between *r*_*tt*_ = 0.81 (adaptive strategies) and *r*_*tt*_ = 0.73 (maladaptive strategies). The secondary scale, called “adaptive scales” for specific emotions shows a very good internal consistency α = 0.88 for sadness, α = 0.83 for anxiety, and α = 0.83 for anger. The maladaptive scale shows internal consistency for anxiety α = 0.59, for sadness α = 0.59 and for anger α = 0.58. Furthermore, “additional scales” provide additional data on the strategies, “expression,” “social support,” and “emotion control,” which are not covered in the two secondary scales. In addition, psycho-social skills and resources are included in this instrument (78).

Experimental Intervention

START (Stress-Arousal-Regulation-Treatment) is a manualized 5-week group-training, based on previous research. It comprises modules within 10 sessions of each 60 min. Each module is highly structured and follows a standard sequence of activities and tasks, including tools for improving mood, reducing maladaptive behavior, and regulation of stress, negative emotions, and tension. START was delivered by one therapist and one nurse twice a week in groups of five to six adolescents with severe dysregulation. The basic concept is derived from DBT, EMDR, and Tf-CBT. All participants obtained the worksheets for each step/module of the treatment program, as well as colorful and playful illustrations.

The manual contains multilingual therapy materials, numerous pictures, and additionally, all information/worksheets as audio files. START favors a play-like and multimedia atmosphere for skills training. The program encourages adolescents to try new skills, all modules are designed interactively and still follow a recurring, clearly understandable structure. A central element of the treatment is the hands on and easy to perform manual construction of an individual skills list as well as encouragement strategies for children and adolescents to discover their own tools for self-regulation with emphasis on individual strengths and resources.

The manual contains worksheets in English, Italian, Arabic, Dari, and German for each step/module of the treatment program. Central characteristic are many colorful illustrations—therefore, the language and attention span requirements are very low. The program has been described in more detail in the START-manual and articles of the authors Dixius and Möhler (66, 70). The treatment does explicitly not contain a narrative approach or exposition-based therapy as this would not be appropriate as a first step for adolescents in highly unstable emotional or psychosocial situations.

### Data Analysis and Statistics

All of the analyses were performed with IBM Statistics SPSS, version 21.0. A dependent *t*-test was applied for comparison of pre- vs. post-treatment raw scores and *t*-values. The mean value comparison of the FEEL-KJ was performed based on normalized *t*-values, taking into account the prerequisites by means of a *t*-test for dependent samples. The hypothesis postulates that the 5-week START- treatment course will show an effect on emotional regulation as indicated by a significant difference in the mean *t-*values pre- and post-treatment. The mean values of the FEEL-KJ were compared with a dependent *t*-test for paired samples. For this pilot trial in order to assess feasibility alpha level was not adjusted.

For the FEEL-KJ, the *t*-values of the major scales, as well as all sub-scales, were used.

While in almost all procedures a higher value is associated with a higher symptom score, the FEEL-KJ must be interpreted on the basis of the *t-*distribution. A value between 25 and 50 is considered as an average for all scales. For *adaptive strategies*, a lower value is considered to be worse while for *maladaptive strategies*, a higher value is considered less desirable.

A per-protocol analysis was conducted in the analysis if they answered all questions of the screenings or finished the post-screening. Furthermore, they were excluded from single questionnaires if items were missing in the respective questionnaire. Therefore, the participants can vary in numbers, depending on the different instruments.

## Results

The frequency of the psychiatric diagnoses of the participants in the START intervention are shown in Table 1.

**Table 1:** Diagnoses of participants.

**Diagnoses**	**Frequency**	**Percent**
Mental and behavioral disorders due to psychoactive substance use	1	2
Depressive disorders	9	14
Anxiety disorders, mixed anxiety and depressive disorders, predominantly obsessional thoughts	3	2
Acute stress reaction, post-traumatic stress disorders, adjustment disorders	40	61
Eating disorders	1	2
Emotionally unstable personality disorders	6	9
Behavioral and emotional disorders with onset usually occurring in childhood and adolescence	6	9

### Child and Adolescent Trauma Scale

The PTSD symptoms in our sample range from 6 to 50 with a mean of 31.97 (*SD* = 12.59).

Forty-five out of 60 patients (75%) scored above the cut off of 21 for clinical relevance of trauma.

### CPTCI—Child and Adolescent Post-traumatic Cognitions Inventory CPTCI

The sample showed results ranging from 26 to 95 in the CPTCI, with a mean of 63.19 (SD = 17.39), based on the cut off between 46 and 48. Clinically relevant symptoms of PTSD can be found in 53 out of 64 participants (83%).

### FEEL-KJ

A significant increase was observed for the adaptive strategies “forgetting,” changing from 39.39 (*SD* = 11.88) to 42.59 (*SD* = 11.88, *t* = −2.01 *n* = 55, *d* = 0.271, *p* = 0.049), “problem solving” changing from 39.59 to 43.86 (*SD* = 12.55, *t* = −2,44, *n* = 55, *d* = 0.325, *p* = 0.018) and “dealing with anger” from 38.54 to 41.84 (*SD* = 12.55, *t* = −2,17, *n* = 55, *d* = 0.309, *p* = 0.034).

A positive trend can be described in all other scales just missing significance (see Table 2).

**Table 2:** FEEL-KJ adaptive scales.

**FEEL-KJ**	**Mean T1**	**Mean T2**	**M_Diff**	**M.Diff_SD**	***t***	**df**	**Sign**.	**Cohens d**
Adaptive strategies total	37.75	40.16	−2.41	14.13	−1.28	55	0.207	0.202
Problem orientated action	36.66	38.77	−2.11	14.04	−1.12	55	0.266	0.176
Distraction	38.45	40.61	−2.16	10.79	−1.5	55	0.14	0.2
Mood improvement	39.11	31.96	−2.86	12.8	−1.67	55	0.101	0.269
Accepting	38.79	42.18	−3.39	14.21	−1.79	55	0.08	0.289
Forgetting	39.39	42.59	−3.2	11.88	−2.01	55	0.049	0.271
Proplem solving	39.59	43.68	−4.09	12.55	−2.44	55	0.018	0.325
Refraiming	44.66	47.18	−2.52	14.59	−1.3	55	0.202	0.271
Dealing with anger	38.54	41.84	−3.3	11.4	−2.17	55	0.034	0.309
Dealing with anxiety	38.32	40.59	−2.27	13.6	−1.25	55	0.217	0.196
Dealing with grief	38.23	40.95	−2.71	13.56	−1.5	55	0.14	0.187

**Figure 1:**
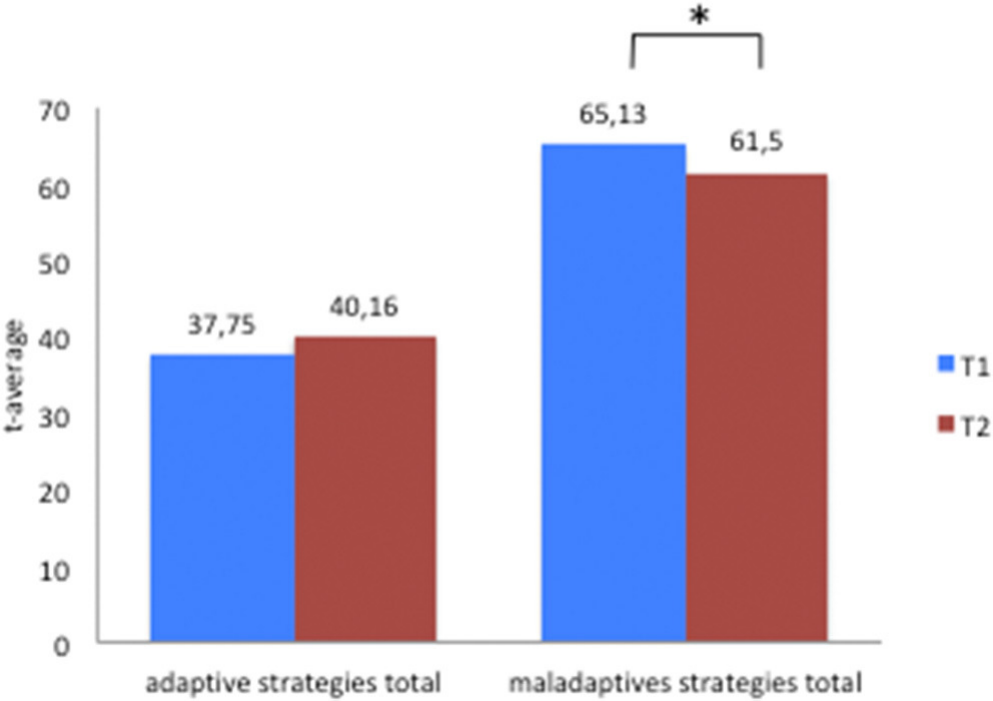
FEEL-KJ-Adaptive and maladaptive scales-total. * symbol of significance.

As illustrated by Figure 1 the change in the total maladaptive strategies before and after the intervention was statistically significant, changing from 65.13 to 61.5 (*SD* = 12.68, *t* = 2.14 *n* = 55, *d* = −0.248, *p* = 0.037). However, the differences between the mean scores for the subscales at baseline and after the intervention were not statistically significant (see Table 3).

**Table 3:** FEEL-KJ maladaptive scales.

**FEEL-KJ**	**Mean T1**	**Mean T2**	**M_Diff**	**M.Diff_SD**	***t***	**df**	**Sign**.	**Cohens d**
Maladaptives strategies **total**	65.13	61.5	3.63	12.68	2.14	55	0.037	−0.248
Aggressive behavior	55.88	55.18	0.7	13.07	0.4	55	0.692	−0.046
Giving up	60.95	59.88	1.07	15.09	0.53	55	0.597	−0.073
Withdrawal	62.59	60.73	2.21	9.34	1.78	55	0.081	−0.181
Self-deprecating	59.02	59	0.02	13.21	0.01	55	0.922	−0.001
Perseveration	52.98	51.14	1.84	13.44	1.02	55	0.31	−0.141
Dealing with anger	62.36	60.16	2.2	13.48	1.22	55	0.228	−0.157
Dealing with anxiety	61.68	59.82	1.86	12.15	1.14	55	0.258	−0.12
Dealing with grief	62	59.63	3.38	12.41	1.43	55	0.158	−0.172

The mean score for adaptive strategies is not statistically significant (see Figure 1), changing from 37.75 to 40.16 (*SD* = 14.13, *t* = −1.28, *n* = 55, *d* = 0.202, *p* = 0.202).

**Figure 2:**
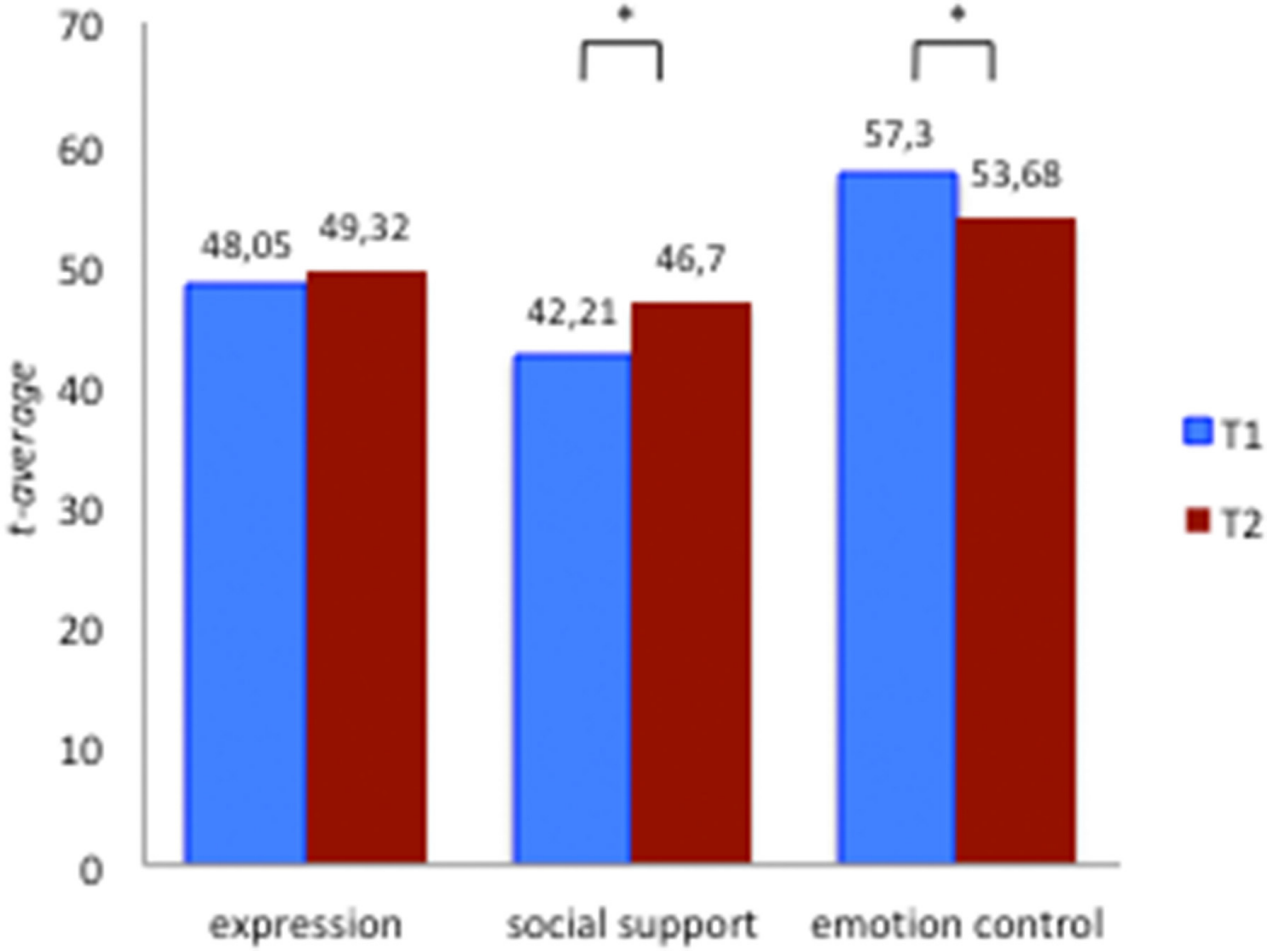
FEEL-KJ additional scales. * symbol of significance.

The results of the additional scales FEEL-KJ “social support” and “emotional control” pre- and post-treatment are presented in Table 4 and Figure 2. Concerning the *additional strategies*, a statistical significant improvement was noticed on *social support* (*SD* = 10.57, *t* = −1.14 *n* = 55, *d* = 4.415, *p* = 0.002) and a reduction for *control of emotions* (*SD* = 10.98, *t* = 2.47, *d* = −0.305, *p* = 0.017) was identified.

**Table 4:** FEEL-KJ additional scales.

**FEEL-KJ**	**Mean T1**	**Mean T2**	**M-Diff**	**M-Diff_SD**	***t***	**N**	**Sign**.	**Cohens d**
Expression	48.05	49.32	−1.27	8.34	−1.14	55	0.260	0.108
Social support	42.21	46.70	−4.48	10.57	−3.17	55	0.002	4.415
Emotion control	57.30	53.68	3.63	10.98	2.47	55	0.017	−0.305

## Discussion

The present study aimed at investigating whether the 5-week structured START-intervention has a positive impact on emotional regulation in adolescents in acute emotional crises. A first result of the study is a history of clinically relevant trauma in about 75% of adolescents referred for acute treatment of symptoms of ED. As these referrals were due to suicidal or highly aggressive behavior our findings underline the general necessity of trauma-informed diagnostic and therapeutic approaches in child and adolescent psychiatry. Specifically, medical guidelines for assessment of child and adolescent psychiatric disorders should all include a through investigation of traumatic or adverse life experiences of the young patient, requiring trauma sensitive care in everyday interaction as well as therapy of the young patient.

Furthermore, this evaluation indicates the feasibility and potential usefulness of START for highly stressed children and adolescents of different nationalities in acute crisis. The largest proportion of the sample showed symptoms of PTSD (75–83%). Almost all adolescents completed the whole therapy program, thereby showing that the program is capable of keeping even highly dysregulated and easily frustrated adolescents with a short attention span and low frustration tolerance motivated and with a good to satisfactory compliance for the program. The results, however, are clearly limited by the size of the sample and the lack of treatment as usual control group.

These preliminary data give first indications of the feasibility and effectiveness of the short-term treatment program START in ED by reducing maladaptive strategies and a trend for increasing the use of adaptive strategies after 5 weeks of treatment. This might indicate a potentially suitable approach to fill a gap in the treatment of adolescents with low treatment motivation and limited frustration tolerance and attention span or severe stress and adverse experiences. Highly unstable adolescents tend to not benefit sufficiently from routine clinical care. Furthermore, some adolescents seem not stable enough to participate in treatment programs with higher thresholds regarding intellect or motivation, i.e., programs such as Dialectic Behavior Therapy (DBT) or Tf-CBT. The low threshold treatment program START presented here might easily be implemented in routine clinical practice and combined with other treatments like DBT or exposure oriented therapies, once a first emotional stabilization has been achieved.

Prominent results are an increase in some adaptive strategies of ER and a decrease of maladaptive strategies for emotional regulation as expected for these variables, constituting the primary target of the 5-week program. Adaptive strategies for ER could contribute to the self-validation of one's own emotions. ER is important for a healthy development (32) and plays a central role in the development of mental disorders. Gross and Thomson (80) postulate, that first of all, it is important to develop ER strategies for then being able to use them contextually.

Regarding the fact that START is a short-term group program in a playful and low threshold manner without narrative elements or trauma exposition, it might be assumed that for a strong, significant, and lasting improvement in general mental health a more profound and individualized therapeutic setting could be necessary. However, more complex and structured approaches such as DBT (11) and Tf-CBT require more behavioral stability than adolescents in acute crises or transition situations are able to display or willing to develop.

Tf-CBT was started after completing START in 21 cases out of our sample as the patients were now found to display enough behavioral stability and therapeutic motivation for a narrative approach after achieving fast success with emotional regulation and self-control. The capability for self-regulation seems to be of utmost importance for successful longterm psychotherapeutic treatments. The capability of handling emotional frustration without the additional necessity for hospitalization, or involuntary constraint, involving potential re-traumatization, is regarded as a major goal of this novel treatment program. However, as emotion regulation is also considered a resilience factor START might be in a second step applied for preventive purposes, e.g., by professionals within the youth welfare system or even in schools.

Limitations of the study exist in the small sample size and the absence of a treatment as usual—control group. Another limitation is that all information was obtained using self-report measures only. A randomized control study including clinician and caregiver report measures is warranted and underway.

These future studies should therefore (a) include a treatment as usual control group (b) use other than self-report instruments also and (c) focus on other settings and include broader assessment tools. Some authors (10) postulate that the challenge for clinicians is to incorporate clinical interventions into non-clinical settings for preventive purposes.

### Clinical Relevance

The capability to handle extreme stress without the additional necessity for hospitalization, or involuntary constraint, involving potential re-traumatization is regarded as a major target of START and a major potential patient benefit. The novel intervention offers emotionally dysregulated adolescents a first aid for crises management and emotional stabilization including skills to reduce self- and other harming behaviors as well as impulsive behavior. START might potentially become a highly useful and cost-saving tool for patients with acute ED because (1) length of inpatient acute treatment might be decreased and (2) hospital re-admissions might be avoided, thus saving mental health or juvenile care costs. Short, simple, inter-cultural programs like START appears to become more and more necessary.

Due to the simple but structured system of the manual, including worksheets in different languages for each step, it could in a second step also be applied by childcare professionals without intense psychotherapeutic background.

In addition to this, self- esteem might potentially be promoted by START, since ER can be regarded as a central aspect of resilience. Several studies have highlighted the significance of ED for the development of psychopathology. Moreover, comorbid severe dysregulation can (1) be the primary cause for admission to psychiatric wards, and (2) pose a significant hurdle for the successful intervention for core symptoms of the primary disorder, such as PTSD. As a consequence, the length of stay on emergency/closed wards is increased, and acute interventions “by force” might lead to re-traumatizations, posing a significant burden on patients and their families, but also on the health care system.

In the long run, early ED in children and adolescents predicts a range of psychiatric, general medical, and social problems in adolescence and young adulthood emphasizing its public health significance. Adolescents with ED experience significant social impairments (e.g., relationship difficulties with parents, siblings, and teachers, school suspension, service use (mental health and general medical), and poverty. An improvement in ED can improve the social and emotional as well as academic development in children and adolescents. The results might support the applicability and efficacy of START with an additional advantage of integration and strengthening of resilience in several populations at risk. This study shows that the 5 weeks low threshold START intervention is being very well-accepted by acutely de-stabilized adolescents and shows a trend to positively influence ED, and therefore potentially stress resilience in adolescents. Future studies in different settings, different age groups and with larger sample sizes are underway.

Our preliminary findings also suggest that the targeted effects warrant further investigation in randomized control trials as well as different settings and populations.

## Data Availability Statement

The datasets are available on request (a.dixius@sb.shg-kliniken.de).

## Ethics Statement

The studies involving human participants were reviewed and approved by the studies involving human participants were reviewed and approved by the Regional Ethics Board of Medical Association Saarland, Germany (No: 189/1). The study was performed in accordance with ethical standards laid down in the Declaration of Helsinki 1964 and its later amendments. All legal guardians gave their informed consent, and children and adolescents provided their informed assent prior to their inclusion in the study. Written informed consent to participate in this study was provided by the participants' legal guardian/next of kin.

## Author Contributions

AD conducted the statistical analysis. EM and AD drafted the first version of the text and EM reviewed and revised the manuscript. All authors contributed to the article and approved the submitted version.

## Conflict of Interest

The authors declare that the research was conducted in the absence of any commercial or financial relationships that could be construed as a potential conflict of interest.

## Abbreviations

ADHD: Attention Deficit Hyperactivity Disorder
BDP: Borderline Personality Disorder
CATS: Child and Adolescent Trauma Screen
CPT: Continuous Performance Test
CPTCI: Child Post-Traumatic Cognitions Inventory
DBT: Dialectical Behavior Therapy
DISYPS III: Diagnostik-System für psychische Störungen
ED: Emotional Dysregulation
EMDR: Eye Movement Desensitization and Reprocessing
ER: Emotional Regulation
FEEL-KJ: Fragebogen zur Erhebung der Emotionsregulation bei Kindern und Jugendlichen
ODD: Oppositional Defiant Disorder
PTSD: Post-traumatic Stress Disorder
START: Stress-Trauma symptoms-Arousal-Regulation-Treatment
Tf-CBT: Trauma-focused – Cognitive Behavioral Therapy.
